# Hepatic and Renal Failure after Anterior Myocardial Infarction Induced Apical Ventricular Septal Defect

**DOI:** 10.1155/2010/645236

**Published:** 2011-01-17

**Authors:** Dirk Lossnitzer, Vedat Schwenger, Stephanie Lehrke, Evangelos Giannitsis, Martin Zeier, Hugo A. Katus, Henning Steen

**Affiliations:** ^1^Division of Cardiology, Pneumology and Angiology, Department of Internal Medicine III, University of Heidelberg, 69120 Heidelberg, Germany; ^2^Division of Nephrology, University of Heidelberg, 69120 Heidelberg, Germany

## Abstract

We report the case of a 68-year-old man suffering from incremental hepatic and renal failure one month after anterior myocardial infarction. Cardiac MRI showed a pronounced apical post-AMI aneurysm, a moderate to severe mitral and tricuspid regurgitation as well as a hemodynamically highly significant 12 mm apical ventricular septal defect with a left-to-right ventricular shunt of almost 63% as the underlying cause. Heart X-ray revealed a severe LAD in-stent restenosis. CAPD catheter drainage of hydroperitoneum due to congestive liver and renal failure was provided in combination with intensified CAPD hemodialysis. Heart surgery was performed where the apical aneurysm was excised, the mitral valve was reconstructed, the IVSD was closed and the subtotally in-stent occluded LAD was bypassed. Post-surgery, the ascites were significantly reduced, and CAPD hemodialysis therapy could be terminated since the renal function gradually improved (MDRD = 25 mL/min). To our knowledge, for the first time we report successful CAPD catheter drainage of hydroperitoneum in combination with CAPD hemodialysis.

## 1. Case Report

**Figure 1:**
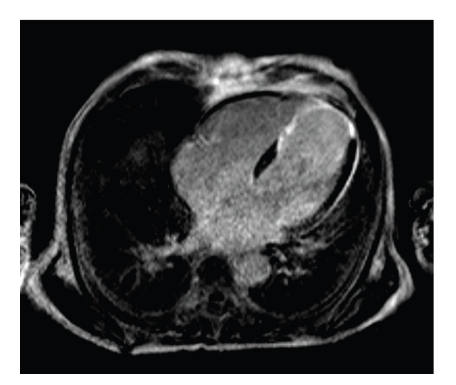
Four-chamber CE-MRI revealed an extensive transmural anterior AMI with septal involvement.

**Figure 2:**
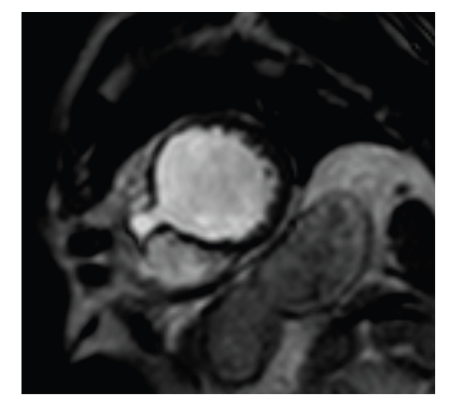
Short axis MRI shows the hemodynamically significant IVSD.

A 68-year-old man who lived in Venezuela for the last four decades presented to our clinic with inexplicably incremental renal and hepatic failure with an obviously reduced general and nutritional condition for further diagnostic and therapy. Due to severely reduced creatinine-clearance (MDRD = 14 mL/min), he was scheduled for continuous ambulatory peritoneal dialysis (CAPD) both to drain his up to six litre nonmalignant, persistent hydroperitoneum as well as for dialysis. Patient's history was inconspicuous apart from a single vessel coronary heart disease that had lead to an acute myocardial infarction (AMI) interventionally treated by implantation of a bare metal stent into the LAD three years ago. One-month post-AMI, the patient complained of progressive abdominal heaviness and pressure as well as shortness of breath, which was nebulously explained by hepatic failure due to thienopyridine treatment. In contrast to the assumed hepatic cirrhosis, multiple abdominal ultrasound studies did not reveal cirrhotic hepatic tissue texture as well as excluded a veno-occlusive Budd Chiari disease. Shortly before the admission to our hospital echocardiography study was conducted externally with reduced acoustic windows to exclude an underlying cardiac disease and showed a moderately reduced left ventricular function, a mild aortic and mitral regurgitation as well as anterior hypo- to akinesia with potential apical adhesions. These findings were in far contrast to a holosystolic harsh loud murmur accompanied by a thrill being present in the third and fourth left intercostal space. For further clarification of morphology, function, the extent of myocardial scar, and exclusion of constrictive pericarditis, a gadolinium contrast enhanced and cine MRI was conducted. Cine short axis, two- and four-chamber MRI imaging showed a dilated right and left ventricle with an almost severely reduced left and right ventricular function (LV ejection fraction = 35%), a pronounced apical post-AMI aneurysm with transmurally infarcted myocardial tissue (Figure 1) as thin as 1.5 mm together with a moderate to severe mitral and tricuspid regurgitation. Short axis imaging (Figure 2) revealed a hemodynamically highly significant 12 mm large apical interventricular septal defect (IVSD) with an MRI estimated regurgitation fraction of 52%. Therefore, having come to the diagnosis of a post-AMI ventricular IVSD, we consequently conducted a left and right heart transseptal catheterisation. Heart X-ray confirmed MRI results and revealed a left to right ventricular shunt of almost 63%, a severe LAD in-stent restenosis, and a severe mitral regurgitation. Despite the administration of more than 180 mL of X-ray contrast agent, patient's renal function was preserved due to an intensified CAPD regime. Heart surgery was performed where the apical aneurysm was excised, the mitral valve was reconstructed, the IVSD was closed and the subtotally in-stent occluded LAD was bypassed with an internal mammaria coronary artery bypass graft. Post-surgery, the ascites were significantly reduced. CAPD therapy could be terminated since the renal function gradually improved (MDRD = 25 ml/min). 

The overall incidence of post-AMI IVSD is hard to assess because clinical and autopsy series differ considerably [1]. A large autopsy study in 1989 suggests that the incidence of myocardial interventricular rupture has increased since the late 1960s, with a rate of 31 per cent among necropsied cases [2]. The prior use of corticosteroids or nonsteroidal anti-inflammatory agents has been implicated as predisposing to rupture as a result of impaired healing. Conversely, the early use of thrombolytic therapy appears to reduce the incidence of cardiac rupture [3], an effect that is responsible in part for improved survival with effective thrombolysis. The size of the defect determines the magnitude of the left to right shunt and the extent of hemodynamic deterioration [4]. The likelihood of survival depends on the degree of impairment of ventricular function and the size of the defect [5]. Recently a non-ischemic surgical repair was described but was not suitable in this patient due to the complex situation this newer approach without aortic occlusion, systemic hypothermia and cardiolplegic arrest. 

In our case, due to right-sided heart failure with increased right atrial pressure and tricuspid regurgitation, subsequent congestive hepatomegaly associated ascites was extensively treated with diuretics and consequently led to hepatorenal syndrome. Although echocardiography might not always suggest IVSD due to reduced acoustic windows, simple and inexpensive auscultation is almost pathognomonic. An IVSD is characterized by the appearance of a new harsh, loud holosystolic murmur at the lower left sternal border and is usually accompanied by a thrill. Although biventricular failure generally ensues within hours to days, in our case the patient astonishingly survived more than three years with accretive signs of hepatic and renal failure. Operative intervention is mostly successful and should not be delayed in patients with a correctable lesion [6].

Our case report has two important issues. To our knowledge, for the first time we report successfully practiced CAPD catheter drainage of hydroperitoneum due to congestive liver and renal failure in combination with CAPD hemodialysis. Secondly, simple auscultation of the heart lead to the diagnosis of IVSD that, if correctly performed earlier, could have greatly relieved the patient's symptoms, his reduced physical state, and his commencing hepatic and renal failure. Furthermore cardiac TEE or MRI as not invasive and radiation-free methods should be considered in case of limited acoustic windows for echocardiography to exclude an IVSD.
